# Machine Learning for the Genomic Prediction of Growth Traits in a Composite Beef Cattle Population

**DOI:** 10.3390/ani14203014

**Published:** 2024-10-18

**Authors:** El Hamidi Hay

**Affiliations:** USDA Agricultural Research Service, Fort Keogh Livestock and Range Research Laboratory, Miles City, MT 59301, USA; elhamidi.hay@usda.gov

**Keywords:** machine learning, genomic prediction, beef cattle

## Abstract

**Simple Summary:**

Genomic selection is commonly used in many livestock species to predict important traits. However, the current methods used to make these predictions are not perfect. New approaches, like machine learning, could make these predictions more accurate because they can handle complex relationships in the data. In this study, we tested machine learning methods, Random Forest, Support Vector Machine, Multi-Layer Perceptron, and Convolutional Neural Networks to predict birth weight, weaning weight, and yearling weight in a beef cattle population. We compared these methods with three traditional ones—GBLUP, BayesA, and BayesB. The GBLUP method was the most accurate for predicting birth and yearling weights, while the Random Forest method was better at predicting weaning weight. Additionally, GBLUP provided a closer match to the actual data. Overall, GBLUP gave better predictions and fit the data better than the machine learning methods we tested.

**Abstract:**

The adoption of genomic selection is prevalent across various plant and livestock species, yet existing models for predicting genomic breeding values often remain suboptimal. Machine learning models present a promising avenue to enhance prediction accuracy due to their ability to accommodate both linear and non-linear relationships. In this study, we evaluated four machine learning models—Random Forest, Support Vector Machine, Convolutional Neural Networks, and Multi-Layer Perceptrons—for predicting genomic values related to birth weight (BW), weaning weight (WW), and yearling weight (YW), and compared them with other conventional models—GBLUP (Genomic Best Linear Unbiased Prediction), Bayes A, and Bayes B. The results demonstrated that the GBLUP model achieved the highest prediction accuracy for both BW and YW, whereas the Random Forest model exhibited a superior prediction accuracy for WW. Furthermore, GBLUP outperformed the other models in terms of model fit, as evidenced by the lower mean square error values and regression coefficients of the corrected phenotypes on predicted values. Overall, the GBLUP model delivered a superior prediction accuracy and model fit compared to the machine learning models tested.

## 1. Introduction

The advent of high-throughput technology and its decreasing cost have allowed for the adoption of genomic selection in most livestock species [1,2,3]. The accuracy of genomic selection is influenced by several factors such as the density of the single nucleotide polymorphism (SNP) panel used, the size of the training population, the genetic relatedness of the validation and training populations, the heritability, and the prediction method employed [4,5]. Currently, the methods predominantly used include Genomic Best Linear Unbiased Predictor (GBLUP); single-step GBLUP (ssGBLUP), which combines genotyped and non-genotyped animals [6]; and Bayesian regression models [7,8]. The primary differences among these approaches generally arise from the assumed distribution of SNP marker effects [9]. These models fail to capture complex non-linear interactions such as dominance and epistasis. Therefore, exploring other prediction models is warranted.

In recent years, machine learning (ML) models have garnered the interests of fields including plant and animal breeding [10,11]. Several studies using ML models showed some improvement in accuracy compared to GBLUP and other Bayesian alphabet models [12,13]. However, their superiority did not extend to all traits and species [14,15]. Therefore, the objective of this study is to compare the performance of ML models with existing genomic prediction approaches using the growth traits of a closed composite beef cattle population.

## 2. Materials and Methods

### 2.1. Data

Data used in this study consisted of 4680 Composite Gene Combination (CGC) animals (½ Red Angus, ¼ Charolais, ¼ Tarentaise; [16]) born between 2002 and 2019 at USDA-ARS, Fort Keogh Livestock and Range Research Laboratory, Miles City, MT. The Pedigree consisted of 9903 animals.

Phenotypes consisted of birth weight (BW), weaning weight (WW), and yearling weight (YW); for further details on the phenotypes and management refer to [17]. Weaning weights were adjusted to 205 days, yearling weights were adjusted to 365 days, and outliers were removed. A summary description of the data is presented in Table 1.

Animals were genotyped using a mixture of low-density SNP 3k panel and high-density Illumina Bovine50k (Illumina, San Diego, CA, USA). Genotypes were called in the Illumina Genome Studio software V2.0.5. Quality control was performed, which consisted of excluding SNP markers with minor allele frequencies of less than 0.05, SNP with a call rate (CRSNP) < 0.90, and Fisher’s exact test *p*-values for Hardy–Weinberg equilibrium < 1 × 10^−5^. After quality control, the number of SNP genotypes consisted of 40,533 SNP markers. Missing genotypes for animals with low-density SNP panel were imputed with FImpute V3.0 software using population and pedigree information [18]. The average allelic R2 was 0.94, which indicates the high imputation accuracy of the missing genotypes.

### 2.2. Statistical Models

In this study, genomic prediction analysis was carried out using corrected phenotypes as the response variable. The fixed effects to correct the phenotypes included sex and contemporary group effect (year of calving and age of dam subclasses). Correction was performed to remove any bias due to the double counting of phenotypic and pedigree information. BLUPF90 software was used [19].

The classical pedigree-based Best Linear Unbiased Predictor (BLUP) using a single-trait model was performed to separately estimate the variance components and breeding values for each trait. The model is as follows:

(1) $$y=Xb+Z_{1}a+Z_{2}m+e,$$

in which **y**, **b**, **a**, **m**, and **e** are the vectors of phenotypes, fixed effects, random additive genetic effects, random maternal genetic effects, and residual effects, respectively; **X**, $Z_{1}$, and $Z_{2}$ are the incidence matrices relating fixed effects, random additive genetic effects, and random maternal genetic effects, respectively, to the observations. For YW, the maternal effect was not included.

#### 2.2.1. GBLUP

The Genomic Best Linear Unbiased Predictor (GBLUP) model is described in [20] and is as follows:

(2) y=Za+e,

where **y** is the vector of corrected phenotypes, **a** is the vector of random animal additive effects, **e** is the vector of random errors, and **Z** is an incidence matrix allocating observations to **a**. The random additive effects were distributed as g~N(0, **G**$\sigma_{g}^{2}$), where **G** is the genomic relationship matrix, and $\sigma_{g}^{2}$ and $\sigma_{e}^{2}$ were the additive genetic variance. Random errors were distributed as **e**~N (0, **I**$\sigma_{g}^{2}$), where $\sigma_{g}^{2}$ is the residual variance. The model was implemented using the BLUPF90 package [19].

#### 2.2.2. Bayes A

The Bayes A model used to estimate SNP effects is as follows:

(3) $$y=\mu +\sum_{j=1}^{n}z_{j}\alpha_{j}+e,$$

where **y** is the vector of corrected phenotypes, µ is the overall mean, n is the number of SNP, $z_{j}$ is the genotype covariate of the jth SNP coded according to the additive model (0, 1, and 2), $\alpha_{j}$ is the allelic substitution effect of SNP_j_, and **e** is the vector of random residuals. A total of 50,000 MCMC iterations with 10,000 burn-in cycles were used. Convergence testing was performed for all parameters following the methods of J Geweke [21] and P Heidelberger and PD Welch [22], and a visual analysis of trace plots was also performed using Bayesian Output Analysis program in R software 4.4.1 (R Development Core Team, Vienna, Austria).

#### 2.2.3. Bayes B

For Bayes B, the model is similar to the Bayes A model in Equation (2), except the SNP effects are modeled as $\sum_{j=1}^{n}z_{j}\alpha_{j}\delta_{j}$, where z_j_ is the genotype of the jth marker, coded according to the additive model (0, 1, and 2); α_j_ is the effect of SNP marker j; and δ_j_ is an indicator variable that is equal to 1 if the jth marker has a non-zero effect on the trait and 0 otherwise. A binominal distribution with known probability π = 0.01 was assumed for δ_j_. For both the Bayes A and Bayes B models, GenSel software (Version 2.14) was used.

#### 2.2.4. Multi-Layer Perceptron

Multi-Layer Perceptron (MLP) is a type of artificial neural network (NN) composed of multiple layers of nodes [23,24,25]. Each node, or neuron, in a layer uses a non-linear activation function, enabling the network to model complex relationships in the data. The architecture typically includes an input layer, one or more hidden layers, and an output layer, making it powerful for both classification and regression tasks, as is the case in this study. MLPs are trained using a supervised learning technique called backpropagation, which adjusts the weights of the connections to minimize the error in the predictions. In this model, two hidden layers were used. The non-linear activation function for the first hidden layer was the rectifier linear, and the soft rectifier linear was applied for the second layer. For more details on the model applied to genomic prediction, please refer to [26,27]. Python V3.10 was used with library TensorFlow and its high-level API, Keras [28,29]. Additionally, a grid search approach was used to find the optimum hyperparameters.

#### 2.2.5. Convolutional Neural Networks

Convolutional Neural Networks (CNNs) are a class of deep learning models. CNNs are designed to recognize patterns in data. They consist of multiple layers, including convolutional, pooling, and fully connected layers, to learn patterns [30]. In this model, we used one input layer (genotypic matrix), one convolutional layer, and one pooling layer. Similarly to MLP, the hyperparameters used were determined through a grid search. Additionally, the Tanh (hyperbolic tangent) activation function was used for the convolutional layer, and the soft rectifier linear unit function was then used in the connected layers.

Similarly to MLP, Python V3.10 was used with library TensorFlow and its high-level API, Keras [28,29], and a grid search approach was used to find the optimum hyperparameters.

#### 2.2.6. Random Forest

Random Forest (RF) is a machine learning tool that constructs multiple decision trees during training [31]. Each tree is built by selecting random samples of the data and making splits based on input features that minimize the mean squares error for regression [32]. The RF model in this study was implemented using Scikit-learn version 1.4 in Python V3.10 [33] and the RandomForestRegressor option, since the growth traits were continuous variables. The RF regression model is as follows:

(4) $$y=\frac{1}{M}\sum_{m=1}^{M}t_{m}(\varphi_{m}\left(y:X\right)),$$

where y represents the response from the Random Forest regression model. Each $t_{m}(\varphi_{m}\left(y:X\right))$ denotes an individual regression tree, and M is the total number of trees within the forest. The prediction process involves traversing each tree with the predictor variables and using the estimated value found at each tree’s terminal node as its prediction. To determine the final prediction for the validation data, the predictions from all trees in the Random Forest were averaged. A grid search was utilized to find the optimal hyperparameter and the maximum depth allowable for the trees. Additionally, a 5-fold cross-validation was conducted for hyperparameter tuning.

#### 2.2.7. Support Vector Machine

Support Vector Machine (SVM) is another machine learning model [34,35]. The objective of the model is to achieve a low prediction error on validation data, given the training data. It can handle both linear and non-linear data, allowing it to manage complex relationships. The SVM model in this study is as follows:

(5) $$y=b_{0}+g{\left(x\right)}^{T}b+e,$$

where **b** is the vector of regression weights, $g{\left(x\right)}^{T}$ is the mapping of the genotypes, $b_{0}$ is the vector of bias, and **e** is the vector of random errors.

In this model, the following cost function was minimized:

(6) $$\underset{b_{0},b}{\underbrace{min}}\frac{1}{2}{\left|\left|b\right|\right|}^{2}+C\sum_{i=1}^{n}V(y_{i}-g{\left(x\right)}^{T}b-b_{0}),\;$$

in which

$$V_{\varepsilon }\left(r\right)=\left\{\begin{array}{c} 0,if\left|r\right|<\varepsilon \\ \left|r\right|-\varepsilon ,\;otherwise \end{array}\right.$$

where $V_{\varepsilon \;}\left(r\right)$ is the ε insensitive loss and C is the cost parameter that influences how strictly the model should fit the training data. A higher value of C enforces the classification rules, minimizing the error in the training but risking overfitting and making the model more sensitive to noise.

Since the optimal selection of C is crucial in the prediction accuracy, a grid search method used for the optimum hyperparameters using a 5-fold cross validation. For more details on this model, we followed a similar approach to that in the work of Long et al. [36]. The SVM model was implemented using Scikit-learn version 1.4 in Python V3.10 [33].

#### 2.2.8. Accuracy

The accuracy of genomic prediction was evaluated using a fivefold cross-validation (5-fold CV) approach, in which 2/3 of the data were randomly split into five groups. For each CV, four of the five groups were defined as the reference population, and the last group was treated as the validation group. First, we calculated the Pearson correlation between the corrected phenotypes and the predicted values. Additionally, prediction unbiasedness was evaluated through the regression of corrected phenotypes on predicted values in the validation dataset. The 5-fold cross-validation scheme was performed 10 times, and the overall prediction accuracy and unbiasedness were derived from the averages of these 10 repetitions.

Furthermore, the mean square error (MSE) was also computed, which offers an assessment of both prediction accuracy and bias; a lower MSE indicates a more accurate model.

## 3. Results and Discussion

The heritability estimates derived from the traditional BLUP model are summarized in Table 1. The heritabilities calculated were 0.38 for birth weight, 0.34 for weaning weight, and 0.28 for yearling weight. Our estimates for birth weight are slightly lower than those reported in [37], where a heritability of 0.41 was found in Angus cattle. However, for weaning weight, our estimate is higher; the authors of [37] estimated a heritability of 0.20, suggesting possible differences in data size and structure, as well as the environmental factors influencing this trait across populations. In terms of yearling weight, our results closely match the heritability of 0.25 reported in [38] in polled Hereford cattle. Such differences in heritability estimates might arise from variations in population structure, sample size, or even methodological approaches across various studies.

Table 2 shows the optimum hyperparameters detected using a grid search for the machine learning models; Table 3 outlines the prediction accuracies for each trait using different models. For birth weight, prediction accuracies ranged from 0.21 to 0.44, with the highest accuracy achieved by the GBLUP model, and the lowest by the Bayes B model. Among the machine learning models, CNNs performed the best, resulting in an accuracy of 0.43. This aligns with prior research indicating the robustness of GBLUP in genomic predictions. For instance, Srivastava et al. [39] demonstrated GBLUP’s superior predictive power over machine learning techniques such as SVM, RF, and extreme gradient boosting in predicting traits like backfat thickness and eye muscle area in Hanwoo cattle. Additionally, two studies [26,40] comparing machine learning models with traditional genomic prediction models showed the superiority of GBLIUP over CNN and MLP.

In the case of weaning weight, prediction accuracies varied between 0.26 and 0.45. Notably, the optimized RF model showed the highest accuracy, outperforming all other models. The optimal RF model featured 300 decision trees, as delineated in Table 2, which exceeds the default 100 trees set by Scikit-learn. Although increasing the number of estimators generally enhances model accuracy, as noted by L Breiman [31], this also incurs greater computational demands. Similar trends were observed for yearling weight, where GBLUP again resulted in the highest prediction accuracy.

Machine learning models demonstrated a noticeable increase in prediction accuracy following hyperparameter tuning (Table 2), highlighting the significance of employing methods like grid search to identify ideal hyperparameters for optimal model performance (Table 3). For the deep learning models, the CNNs performed better than MLP for all growth traits. This is consistent with the literature, in Holstein cattle, where the genomic prediction accuracy for sire conception rate was 12% higher for CNNs compared to MLP [26].

Generally, machine learning models achieved prediction accuracies that are competitive with traditional models, except in the case of weaning weight, where the RF model excelled. Such findings are corroborated by the authors of [41] in their evaluation of machine learning models for genomic prediction in pig datasets, revealing a comparable performance across different methodologies. However, divergences exist, as illustrated by the authors of [13], who reported an improved prediction accuracy for reproductive traits with the use of machine learning models. Model performance was also assessed using MSE as a goodness-of-fit metric (Table 4). Typically, a lower MSE indicates a better model fit. In our study, the RF and SVM models resulted in a higher MSE for both birth and yearling weights, while GBLUP consistently presented the lowest MSE. Interestingly, for weaning weight, the RF model achieved the lowest MSE. These results align with the study in [39],which showed GBLUP to have the lowest MSE in genomic predictions. Meanwhile, the authors of [13] found that both SVM and RF offered the lowest MSE for predicting reproductive traits in pigs.

Finally, assessing the inflation or deflation of predicted values through a regression of corrected phenotypes on predicted values within the validation dataset (Table 4), GBLUP emerged as the best model for birth weight, weaning weight, and yearling weight.

## 4. Conclusions

In this study, machine learning models proved to be beneficial in improving prediction accuracy for weaning weight. However, this improvement did not extend to other traits, and the GBLUP model performed better. This limitation is potentially associated with the genetic architecture and the complexity of the trait. Therefore, future efforts are warranted to refine machine learning models for genomic prediction.

## Figures and Tables

**Table 1:** Estimated heritabilities and summary statistics of the growth traits analyzed.

Trait	n	h^2^ (SE)	Mean, kg	SD, kg
BW (kg)	4660	0.38 (0.04)	32.73	4.13
WW (kg)	4651	0.34 (0.09)	204.69	27.70
YW (kg)	4563	0.28 (0.07)	266.87	35.70

**Table 2:** Optimum hyperparameters for SVM and RF models through grid search approach.

Model	Optimum Hyperparameters ^1^
SVM	kernel = ‘rbf’; C = 10; gamma = 0.01
RF	n_estimators = 300; max_depth = none; optimal number of features = 1/3 of total features
MLP	Optimization algorithm = stochastic gradient descent; epochs = 50; learning rate = 0.01; neurons for the first hidden layer = 96; neurons for the second hidden layer = 64
CNN	Optimization algorithm = stochastic gradient descent; epochs = 50; learning rate = 0.01; neurons for the first hidden layer = 32; neurons for the second hidden layer = 16

^1^ The optimal hyperparameters detected through grid search.

**Table 3:** Prediction accuracies of different models for all three growth traits.

Model *	BW	WW	YW
GBLUP	0.447 ± 0.023	0.373 ± 0.019	0.321 ± 0.022
Bayes A	0.426 ± 0.018	0.375 ± 0.012	0.303 ± 0.013
Bayes B	0.21 ± 0.010	0.26 ± 0.022	0.22 ± 0.015
RF_default	0.350 ± 0.025	0.405 ± 0.003	0.261 ± 0.001
SVM_default	0.334 ± 0.014	0.360 ± 0.021	0.280 ± 0.027
RF_optimized	0.421 ± 0.039	0.451 ± 0.011	0.306 ± 0.028
SVM_optimized	0.406 ± 0.034	0.380 ± 0.018	0.311 ± 0.004
MLP	0.408 ± 0.021	0.402 ± 0.027	0.304 ± 0.014
CNN	0.433 ± 0.034	0.419 ± 0.024	0.303 ± 0.011

* Prediction accuracy of the model is calculated as Pearson’s correlation between corrected phenotypes and direct genomic values in the validation dataset.

**Table 4:** Mean square error and regression slope coefficients of different models for all growth traits.

Model	BW		WW		YW	
	β1 ^1^	MSE ^2^	β1 ^1^	MSE ^2^	β	MSE ^2^
GBLUP	0.971 ± 0.122	0.821	0.991 ± 0.107	2.892	0.954 ± 0.138	10.26
Bayes A	0.917 ± 0.130	0.843	1.101 ± 0.125	2.936	0.923 ± 0.137	11.27
Bayes B	0.902 ± 0.109	0.925	0.927 ± 0.117	3.034	0.906 ± 0.122	12.98
RF_default	1.055 ± 0.115	0.911	1.175 ± 0.129	3.071	1.209 ± 0.104	13.12
SVM_default	1.080 ± 0.136	0.906	1.202 ± 0.122	3.280	1.192 ± 0.117	12.15
RF_optimized	1.112 ± 0.127	0.860	1.209 ± 0.130	2.331	1.291 ± 0.102	11.18
SVM_optimized	1.204 ± 0.132	0.872	1.226 ± 0.142	2.714	1.146 ± 0.096	10.34
MLP	1.115 ± 0.132	0.901	1.188 ± 0.119	3.516	1.123 ± 0.116	12.09
CNN	1.102 ± 0.104	0.856	1.031 ± 0.107	3.104	1.101 ± 0.120	11.27

^1^ Regression slope of corrected phenotypes on predicted values for animals in the validation dataset to measure the inflation of genomic prediction. ^2^ Mean squared error of predicted genomic values and corrected phenotypes in the validation dataset as a measure of the fit of the model.

## Data Availability

The data supporting the findings of this study are available upon request from the author El Hamidi Hay: elhamidi.hay@usda.gov and with permission from the USDA Agricultural Research Service.
